# Revisiting the HPLC-FLD Method to Quantify Paralytic Shellfish Toxins: C3,4 Quantification and the First Steps towards Validation

**DOI:** 10.3390/toxins14030179

**Published:** 2022-02-27

**Authors:** Joana F. Leal, Maria L. S. Cristiano

**Affiliations:** 1 Centre of Marine Sciences (CCMAR), University of Algarve (UAlg), Campus de Gambelas, 8005-139 Faro, Portugal; jfleal@ualg.pt; 2 Department of Chemistry and Pharmacy, Faculty of Science and Technology (FCT), University of Algarve (UAlg), Campus de Gambelas, 8005-139 Faro, Portugal

**Keywords:** saxitoxins, C3,4, monitoring, validation, quantification

## Abstract

Paralytic shellfish toxins (PSTs) are a large group of biotoxins that cause paralytic shellfish poisoning. Their appearance in natural waters and their ingestion by aquatic species have a huge socio-economic impact, whereby their monitoring is of the upmost relevance to minimize the consequences. For earlier detection and faster response/action by stakeholders, validation of adjusted analytical methods, particularly for lower concentration levels, is important. This work proposes a derived High-Performance Liquid Chromatography method, with fluorescence detection (HPLC-FLD). The main differences from the official method are the size of the HPLC column and the gradient elution conditions. It covers the current eleven certified reference materials (CRM) available on the market, including the most recent—C3,4. This first calibration report for C3,4 suggests limits of detection (LOD) and limits of quantification (LOQ) of 6 nM and 19 nM (~5 µg STX.2HCl eqv./kg and 17 µg STX.2HCl eqv./kg), respectively. For the remaining CRM, LODs ranged between 3 and 28 nM (~0.9 and 127 µg STX.2HCl eqv./kg), while LOQs varied between 11 and 94 nM (~3 and 409 µg STX.2HCl eqv./kg, considering toxicity equivalency factors (TEFs) reported by EFSA).

## 1. Introduction

Paralytic shellfish toxins (PSTs) are a numerous and complex group of biotoxins [1] produced by dinoflagellates (e.g., *Gymnodinium* spp. and *Alexandrium* spp.). The appearance of such PST-producing dinoflagellates in the natural aquatic environment has major economic and social impacts [2,3]. It directly affects the economy, through the loss of income for producers and catchers (and other players along the sales chain) of the affected aquatic species. Social impacts are reflected in reduced availability of animal protein (caused by the ban on harvesting) and health problems resulting from the ingestion of contaminated food (e.g., shellfish). As such, the availability of accurate, reliable and affordable methods for the detection and quantification of PSTs is of upmost relevance.

Over the years, several methods [4,5,6,7,8] have been developed and/or optimized, aiming to improve analytical parameters (e.g., sensitivity and specificity). Most of them require mass spectrometry techniques, implying higher costs than the methods currently in use (e.g., AOAC Official Method 2005.06 [9,10]). For many laboratories and institutions, it is not easy to obtain funding for the acquisition and maintenance of the equipment and materials necessary to implement such methods. Instead, for monitoring purposes (routine application), the strategy may involve streamlining analytical procedures. For instance, it may be possible to set thresholds, for which it may or may not be useful to conduct the analysis [11]. Another strategy could be to develop validation studies for adjustments to the recommended method, allowing more laboratories to conduct the analyses. This will certainly allow a quicker response to shellfish farmers or catchers who need to know, almost in real time, whether they can capture and sell their products (for direct consumption). Nevertheless, if the objective is the scrutiny of all toxins and the determination of the toxicological profile associated with each condition (e.g., species, bloom, geography), or other more detailed studies, the option of more advanced analytical methods should be considered, whenever possible.

In the course of our work, we encountered some difficulties in implementing the official method (AOAC Official Method 2005.06 [9,10]), mainly as the chromatographic column we have has different characteristics from the one used in the official method. This led us to develop and optimize a derived method, also involving pre-chromatographic oxidation and fluorescence detection. For that, a series of experiments were carried out to evaluate the suitability of the method. We used the eleven certified reference standards (STX; dcSTX; dcGTX2,3; C1,2; C3,4; GTX1,4; GTX2,3; GTX5; GTX6; NEO; dcNEO) currently available on the market. This also means that, to the best of our knowledge, this is the first record of a direct analysis using a CRM for the C3,4 toxins. Until now, these toxins were quantified indirectly by acid hydrolysis of a post ion-exchange solid phase extraction (SPE) fraction [11,12]. Moreover, this work summarizes the pre- and post-analytical (quantification) procedures for each toxin, considering the retention times of our method. We believe that our contribution is important for other researchers in this area and, above all, will improve the capacity to monitor PSTs levels using different methods. 

## 2. Results

### 2.1. Method Development and Validation

Compared to the AOAC Official Method 2005.06, the HPLC (High Performance Liquid Chromatography) column used in this work is longer (by 25 cm). This led to an adjustment of the elution conditions, to reduce the elution time of the analytes. Replacement of the mobile phase B by acetonitrile (CH_3_CN) (in this work) and adjustment of gradient conditions allowed a reduction in the elution times. 

For the method development and its validation, we considered the guidelines of the International Council for Harmonization (ICH) [13]. The following parameters were evaluated: range, linearity, limit of detection (LOD) and limit of quantification (LOQ), precision (instrumental and intermediate precision) and selectivity. The results obtained for these parameters are presented in Table 1, Table 2 and Table 3.

### 2.2. Range and Linearity

The least squares method was applied to the calibration curves of chromatographic peak area (*y*) for each toxin against the toxin concentration (*x*), in µM (*y* = m*x* + b). m is the slope and b is the intersection on the *y*-axis. The linearity of the analytical method is evaluated by the correlation coefficient, *r* (and determination coefficient, *R*^2^), for each toxin. All *r* and *R*^2^ are higher than 0.9990 and 0.9980, respectively, corroborating good linearity for the range of concentrations tested. For each toxin, the lowest concentration of the linear range is always equal or greater than the correspondent LOQ.

### 2.3. LOD and LOQ

LOD and LOQ for each toxin were calculated based on the corresponding calibration curves, as (3σ)/*m* and (10*σ*)/*m*. *σ* is the residual standard deviation of the regression line and *m* is the slope of the regression line [13,17]. LOD ranged between 0.003 µM (C1,2 toxins) and 0.03 µM (STX). LOQ varied between 0.01 µM (dcGTX2,3; C1,2; GTX2,3; GTX5) and 0.09 µM (STX). In general, lower LOD and LOQ values were obtained for the non-*N*-hydroxylated analogues (exception for STX), compared to the *N*-hydroxylated toxins. The TEFs reported by EFSA [18] and the FAO/WHO [19] were included in Table 2. The conversion from µM to µM STX eqv. and to µg STX.2HCl eqv./kg were made according to Equations 1 and 2 [14]. *i* represents each individual toxin; MW is the molecular weight of saxitoxin dihydrochloride (372.2 g/mol); V_E_ is the extract volume (mL); m_H_ is the weight of homogenized tissue (g); D_f_ is the dilution factor of solid-phase extraction (SPE) procedure. To convert the LOQ and LOD concentration units, the values set out in the official method were used (Supplementary Information Figure S1–S3).

(1) $$C\left(\mu M\;\mathrm{STX}\;\mathrm{eqv}.\right)={\mathrm{TEF}}_{i}\times C_{i}\left(\mu M\right)$$

(2) $$C\left(\mu g\;\mathrm{STX}.2\mathrm{HCl}\;\mathrm{eqv}./\mathrm{kg}\right)=C\left(\mu M\;\mathrm{STX}\;\mathrm{eqv}.\right)\times \mathrm{MW}\left(\mathrm{STX}.2\mathrm{HCl}\right)\times \frac{V_{E}\left(\mathrm{mL}\right)}{m_{H}\left(g\right)}\times D_{f}$$

Comparing our LOQs (using TEFs proposed by EFSA) with those obtained with the AOAC official method 2005.06, two different scenarios are observed. Higher values were obtained for STX, NEO, GTX1+4 and dcSTX. Lower values were achieved for dcGTX2+3, GTX2+3, C1+2, C3+4 and GTX5. The biggest difference is observed for the C3,4 toxins: our LOQ value (17 µg STX.2HCl eqv./kg) is ~43 times lower. This major difference could be partially explained by the direct quantification of these toxins, rather than indirect quantification (by hydrolysis). For dcNEO and GTX6, LOQ values are not reported in AOAC 2005.06. 

### 2.4. Precision

To assess the precision of the method, two measurements were considered: instrumental precision and intermediate precision (within-laboratory variations) [13,20]. For instrumental precision, peak areas were assessed from the repeat analysis of toxin standard solutions in one analytical batch (three injections/each calibration level/toxin or mixtures of toxins). Intermediate precision was determined by analyzing freshly prepared standard solutions on different days, as well as freshly prepared reagents and eluents. For the same toxin or mixture of toxins, the minimum and maximum time between calibration replicates was two weeks and two months, respectively. The relative standard deviations (RSD) of these parameters, in percentage, were calculated using Equation (3), where *s* is the standard deviation and $\bar{x}$ is the arithmetic mean. For instrumental precision, RSD ≤ 3.0% were achieved, except for a single calibration point on only one of the replicates (GTX6 or B2 toxin). For intermediate precision, RSD ≤ 22.2% were obtained. Both precision measures meet the minimum performance criteria defined [14]. Additionally, the Horwitz ratio or value (HorRat) was determined to evaluate the acceptability of the analysis method regarding the precision. It is widely used in inter-laboratory studies, however may also be applied to within-laboratory relative standard deviation, albeit with less reliability [20]. For each toxin, the highest RSD (%) for intermediate precision and the correspondent toxin concentration were considered to estimate the Horwitz values, according to Equations (4) and (5). PRSD is the predicted RSD and considers the concentration of each toxin expressed as g STX.2HCl eqv./g. The HorRat values for intermediate precision ranged between 0.1 (C1,2) and 1.7 (dcNEO). Most of our values fall within the limits for performance acceptability (0.5–2.0). As these are data obtained from a single laboratory, HorRat values are expected to be lower than those involving other laboratories [20]. Detailed results are shown in Table 3.

(3) $$\mathrm{RSD}=100.s/\bar{x}$$

PRSD = 2.C^−0.15^ (4)

HorRat = RSD/PRSD(5)

### 2.5. Selectivity

**Figure 1:**
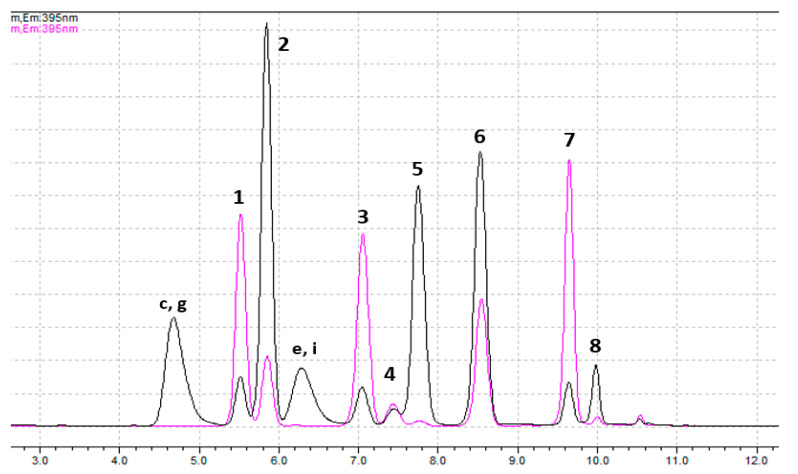
Chromatograms of a mix solution containing 11 CRMs, previously oxidized with periodate (black) or peroxide (pink). Identification of peaks with peroxide oxidation: 1—dcGTX2,3 (1st peak); **2**—dcGTX2,3 (2nd peak); **3**—C1,2; **4**—dcSTX (1st peak); **5**—dcSTX (2nd peak); **6**—GTX2,3; **7**—GTX5; **8**—STX. Identification of peaks with periodate oxidation: 1—dcGTX2,3 (1st peak); **2**—GTX1,4 + C3,4 + dcGTX2,3 (2nd peak); **3**—C1,2; **4**—dcNEO (1st peak); **5**—NEO + GTX6 + dcSTX (2nd peak) + dcNEO (2nd peak); **6**—GTX2,3 + products **d** and **h** (Figure S2) of GTX1,4 and C3,4, respectively; **7**—GTX5; **8**—STX + products **f** and **j** (Figure S2) of NEO and GTX6, respectively. (**c**, **g**, **e** and **i** correspond to the secondary products identified in Figures S1 and S2, in SI).

In addition to the mixtures of standards described in Section 4.2 (Figures S1 and S2), a mixture of all standards was prepared, to better simulate a real mixture of biotoxins. Aliquots of this mixture were used for oxidation with periodate or peroxide and the samples were further analyzed. The corresponding chromatograms are shown in Figure 1 and show good separation of the chromatographic peaks. Note that for quantification of dcGTX2+3, dcSTX and dcNEO the first peaks are used, while the quantification of GTX1+4, C3+4, GTX6 and NEO relies on the second peaks. Analysis of the chromatograms shows that this chromatographic method is selective for toxins dcGTX2,3 (1st peak), C1+2, dcSTX (1st peak), GTX2+3, GTX5 and STX (non-hydroxylated toxins at N1 [1]), using pre-oxidation with peroxide. Also, it is selective for toxins dcGTX2,3 (1st peak), C1+2, dcNEO (1st peak) and GTX5, using pre-oxidation with periodate. Among them, only dcNEO (*N*-hydroxylated toxin) is quantified with this pre-oxidation method. The remaining toxins have a much higher signal in the pre-oxidation with peroxide, therefore this should be the quantification method. 

### 2.6. Quantification of Each PST

Our method co-elutes different toxins at the same retention times (R_t_), namely, GTX6 and NEO, GTX1+4 and C3+4, like the Official method. In the Official method the peaks used for the quantification of GTX6 and NEO, and GTX1+4 and C3+4 appear to elute at ~6 min and ~3 min, respectively. In our method such elutions occur at 7.7–7.8 min and at 5.8–5.9 min, respectively. To solve these issues, in natural samples (e.g., phytoplankton or shellfish) additional procedures to separate biotoxins for their further quantification are required, namely the solid-phase extraction (SPE) using C18 cartridges and COOH ion exchange cartridges, the latter procedure yielding three fractions [9,10]. 

Table 4 presents the retention times for each toxin and its by-products, using our chromatographic method. As can be seen, there is a good precision regarding the R_t_, since throughout the experiments their variation was never greater than 0.2 min. Also, Table 4 details the co-elution of different oxidation products that may interfere with quantification of specific toxins in a mixture and compiles the procedures to be adopted for the quantification in natural samples. 

At R_t_ 5.8–5.9 min (peak 2), there is a co-elution of GTX1+4, C3+4 and dcGTX2+3 (2nd peak). Using SPE-COOH, it is possible to separate C3,4 from the others, as they come out in different fractions. However, in periodate oxidation, the presence of dcGTX2,3 in a real sample may interfere with the quantification of GTX1,4, mainly as the 2nd peak of dcGTX2,3 in periodate oxidation is more intense than with peroxide (Figure S3). When the presence of dcGTX2,3 is not detected in the peroxide oxidation, the peak at this R_t_ will most likely correspond only to GTX1,4. However, if the presence of dcGTX2,3 is detected with peroxide, the contribution of the second peak of dcGTX2,3 should be discounted. Some authors have proposed to plot a graph of dcGTX2,3 areas (oxidized with periodate) versus dcGTX2,3 concentrations. As the dcGTX2,3 concentration is known (previously determined by peroxide oxidation), they propose a deduction of the dcGTX2,3 peak area as an oxidation product of periodate and then its subtraction from the total peak area [21]. Nevertheless, we believe that the approach presented in the AOAC Official Method 2005.06 [9,10] for quantification of NEO (after periodate oxidation) in the presence of dcSTX may also be applied to this situation. Thus, the contribution of dcGTX2,3 (2nd peak) to the quantification of GTX1,4 could be discounted, based on the mathematical ratios of the peaks after oxidation with periodate and peroxide. 

At R_t_ 7.3–7.4 min (peak 4), there is a co-elution of dcSTX and dcNEO. While dcSTX must be quantified after peroxide oxidation, dcNEO must be quantified after periodate oxidation, either after SPE-C18 [11,21] or SPE-COOH-fraction 3 [8]. The chromatograms presented in Figure 1 do not show a significant contribution of dcSTX to the quantification of dcNEO since, at this R_t_, the peak area after periodate oxidation is similar to the peak area after peroxide oxidation (attributed only to dcSTX). Although the figure presented in the AOAC Official Method 2005.06 suggests that the first peak of dcSTX is almost undetectable after oxidation with periodate (in agreement with our results), in the extension of the method [15] the authors claim that dcNEO (1st peak) may also include the contribution of dcSTX (1st peak), which must be subtracted, running a calibration curve of dcSTX standards after periodate oxidation.

At R_t_ 7.7–7.8 min (peak 5), NEO, GTX6, dcSTX (2nd peak) and dcNEO (2nd peak) co-elute. The SPE-COOH fractioning allows the separation of GTX6 from the others. The recommended methodology to quantify NEO requires SPE-C18—SPE-COOH (fraction 3) procedures, followed by periodate oxidation. However, the 2nd peaks of dcSTX and dcNEO may also be detected in the same fraction [21], after periodate oxidation, interfering with NEO quantification. Thus, as for the previous procedures, additional calculations must be made to correctly quantify the NEO. As mentioned above, the AOAC Official Method 2005.06 already presents two methods for quantification of NEO in the presence of dcSTX, both based on the mathematical ratios of the peaks after oxidation with periodate and peroxide. To discount the contribution of dcNEO (2nd peak), the same principle could be adopted (if its presence is previously detected).

At R_t_ 8.4 and 10.0 min (peaks 6 and 8), the presence of by-products from *N*-hydroxylated toxins do not interfere with quantification of GTX2,3 or STX by oxidation with peroxide. 

The by-products that appear at R_t_ 4.6–4.7 min and 6.2–6.4 min (after oxidation with periodate) do not interfere with the quantification of the main toxins.

## 3. Discussion

The detection and quantification of biotoxins in molluscs at low concentrations allows earlier intervention to minimize impacts (e.g., avoiding the unintentional sale of products containing high levels of biotoxins and their subsequent removal) or, in some circumstances, through actions to counteract the spread of the algal bloom. In fact, some authors suggest the importance of implementing warning systems and propose monitoring programs include the monitoring of phytoplankton [22]. 

The main differences from this work and the AOAC Official Method 2005.06 are the size of the HPLC column and the gradient elution conditions. Our results revealed good linearity for all toxins. Also, lower LOQs were achieved for dcGTX2+3, GTX2+3, C1+2, C3+4 and GTX5 toxins, even though the range of concentrations tested is partially coincident with that of the official method. The greatest differences are observed for C1+2 and C3+4 toxins, for which the LOQ values are about 28 and 43 times lower than those of the reference method. Precision measures (peak areas, R_t_, intermediate precision) also meet the minimum performance criteria defined [14]. 

As previously mentioned, it is important to have quick and less expensive methods for routine analysis (monitoring), however the identification of toxins must be clear and unambiguous, to avoid false results. The AOAC 2005.06 Standard Operating Procedure states that when there is no fractionation (separation), the co-eluting toxins must be identified as the individual toxin with the higher toxicity (higher TEF—toxicity equivalency factor). This means that GTX6 and NEO toxins would be identified only as NEO, C3+4, GTX1+4 and dcGTX2+3 only as GTX1+4, dcSTX and dcNEO only as dcNEO [14]. However, this assumption may lead to toxicity values higher than the true values. While the precautionary principle is always preferable from a human health point of view, overestimation of toxicity in the natural samples may prevent the sale of products and cause unnecessary economic repercussions on producers/catchers and sellers of the affected aquatic species. Thus, to obtain more accurate values for each toxin, fractionation by SPE-COOH is preferred, whenever possible (and necessary). In specific cases where the co-elution of the toxins is verified even with fractionation, discounting the contribution of one of them to the quantification of the other should be done cautiously, accounting for possible interferences of the matrix. If, for water-diluted standards, the mathematical ratios between peak areas seem to be an adequate procedure [21], when in a mollusk matrix the ratios of peaks in water may not be the most accurate [23]. The presence of naturally fluorescent components in the matrix should not be overlooked, so it is important to analyze both non-oxidized and oxidized samples and assess their behavior [24].

It is of upmost importance to continue these studies with natural samples, to fully validate the method for the quantification of toxins in shellfish. In fact, some authors [24] have shown the effect of natural matrices in the quantification method, especially in the detector response. Also, the contribution of this effect in different toxins appears to be different [24]. Nevertheless, although more studies are needed, we believe that these preliminary results represent an important contribution to the area. In addition to the first known results with a CRM of C3,4 toxins, we disclose the first steps towards validating this method. These and other adjustments to the official method (duly validated) may increase the capacity of entities to respond to shellfish producers and catchers.

## 4. Materials and Methods

### 4.1. Reagents and Materials

Certified reference standard solutions of STX, dcGTX2+3, C1+2, C3+4, GTX1+4, GTX2+3, GTX5, GTX6, NEO and dcNEO were obtained from CIFGA laboratory S.A. (Lugo, Spain), while a certified reference standard solution of dcSTX (CRM-dSTX-b) was purchased to the National Research Council Canada (Halifax, Canada). The volume of each certified reference material (CRM) ampoule was quantitatively transferred and distributed by different glass recipients (vials), that were stored in frozen conditions (below—18 °C) until required. From each vial, different intermediate solutions were prepared using ultrapure water obtained using a Simplicity^®^ Water Purification System (Merck, Darmstadt, Germany, 18.2 MΩ·cm). 

Pro-analysis (p.a.) reagents (NaOH, Na_2_HPO_4_.2H_2_O) from Chem-Lab, H_5_IO_6_ (≥99.0%) from Sigma-Aldrich, and NH_4_HCO_2_ LC-MS grade from Carlo Erba were used to prepare the aqueous solutions, using ultrapure water. Glacial acetic acid (HPLC grade, ≥99.8%), from Carlo Erba, were used to stop oxidation reactions and to prepare diluted CH_3_COOH solutions (1 M, 0.1 M, 0.1 mM). Acetonitrile (CH_3_CN) HPLC grade (≥99.9%) from Honeywell was used as eluent. PVDF membrane filters (0.22 µm, 47 mm) from Teknokroma were used to filter the solvents, prior to HPLC analysis. 

### 4.2. Calibration Curves

To create the calibration curves, 4–6 concentration levels of each toxin or mixtures of toxins were considered. The standard solutions were prepared in acetic acid (CH_3_COOH) 0.1 mM, from the intermediate solutions. First each toxin was analyzed individually, then mixtures of certified standards of toxins were analyzed. Mix I: dcGTX2,3 + C1,2 + dcSTX; mix II: GTX2,3 + GTX5; STX; mix III: GTX1,4 + NEO; mix IV: C3,4 + GTX6; and dcNEO. The concentrations of each toxin in solution, separately or in the mixture, ranged from 0.01 µM to 1.25 µM. Three replicates of the injection were carried out, for each concentration level. At least three independents (*n* ≥ 3) calibration curves were constructed, in different days, for each toxin or for mixture of toxins. 

### 4.3. Pre-Oxidation Reactions

Before analysis, each standard solution containing one or more toxins was exposed to oxidation conditions with periodate (GTX1+4, C3+4, dcNEO, NEO, GTX6) or peroxide (dcGTX2+3, C1+2, dcSTX, GTX2+3, GTX5, STX). The periodate oxidant was freshly prepared each day of analysis, mixing equal volumes of H_5_IO_6_ (0.03 M), NH_4_HCO_2_ (0.3 M), Na_2_HPO_4_ (0.3 M) and adjusting the pH of the mixture at 8.2 with NaOH 0.2 M, as described in the official method [9,10]. A peroxide (H_2_O_2_) solution (50% *w*/*w*), from Scharlau, was used to prepare H_2_O_2_ solutions 10% (*w*/*v*) for oxidation reactions. The oxidation reactions before analysis were carried out according to the AOAC Official Method 2005.06, with some adaptations. For peroxide oxidation, 375 µL NaOH 1M and 37.6 µL H_2_O_2_ 10% were mixed in an autosampler vial. Then, 150 µL of standard solution were added, the mixture was stirred and let to react at room temperature for 2 min. Lastly, 30 µL of glacial acetic acid were added (a vortex was used for the mixes). For periodate oxidation, 100 µL of ultrapure H_2_O and 100 µL of standard solution were mixed, also using an autosampler vial. After that, 500 µL of periodate oxidant were added and mixed using a vortex. The solution was let to react for 1 min at room temperature and, finally, 5 µL glacial acetic acid were added, mixing well. At least 10 min should be allowed before injection, also at room temperature [9,10].

### 4.4. Method Description and Equipment

Quantitative analysis of PSTs was performed using High-Performance Liquid Chromatography, with fluorescence detection (HPLC-FLD). This equipment (model Prominence-i LC-2030C Plus), from Shimadzu, consists of a quaternary pump, a column oven, a refrigerator autosampler and a spectrofluorometric detector RF-20A XS. A reversed-phase C18 column (Mediterranea Sea18) with dimensions 25 cm x 0.46 (5 µm particle size), together with an ultraguard^TM^ column (Sea18 10 × 3.2 mm), all from Teknokroma, were used. The temperatures of the autosampler, column oven and cell detector were 10 °C, 25 °C and 30 °C, respectively. For the elution of the PSTs oxidation products, two mobile phases were used: A—ammonium formate 0.1 M adjusted to pH 6 with 0.1 M CH_3_COOH; B—CH_3_CN. The elution occurs in gradient mode under the following conditions: 1–5% CH_3_CN in the first six minutes, 5–28% CH_3_CN between 6 and 13 min, 28–1% CH_3_CN between 13 and 16 min, and maintain 1% CH_3_CN for 3 min before the next injection. The flow rate is 1.5 mL/minute. The excitation and emission wavelengths were 340 nm and 395 nm, respectively. The injection volume was 100 µL or 30 µL, for solutions oxidized with periodate or peroxide, respectively.

### 4.5. Statictical Analysis

All data analysis was performed using Microsoft Excel (version 2201). Linear regression was applied to calculate the coefficient of determination, *R*^2^, based on data concentrations (*x*-axis) and the correspondent peak areas (*y*-axis).

## Figures and Tables

**Table 1:** Concentrations range for each toxin, in µM (number of independent replicates for each toxin, *n* ≥ 3); correlation coefficient (*r*); coefficient of determination (*R*^2^); limits of detection (LOD); limits of quantification (LOQ).

PST	Linear Range (µM)	*R* (≥)	*R*^2^ (≥)	LOD (µM)	LOQ (µM)
dcGTX2,3	0.03–0.91	0.9999	0.9998	≥0.004	≥0.01
GTX1,4	0.10–0.78	0.9996	0.9991	≥0.02	≥0.05
C3,4	0.02–0.65	0.9995	0.9994	≥0.006	≥0.02
C1,2	0.01–0.67	1.0000	0.9999	≥0.003	≥0.01
dcSTX	0.08–1.25	0.9997	0.9995	≥0.005	≥0.02
dcNEO	0.07–1.17	0.9997	0.9994	≥0.02	≥0.07
NEO	0.06–1.01	0.9996	0.9992	≥0.02	≥0.06
GTX6	0.05–0.81	0.9999	0.9999	≥0.005	≥0.02
GTX2,3	0.03–0.72	0.9999	0.9998	≥0.004	≥0.01
GTX5	0.03–0.86	1.0000	0.9999	≥0.004	≥0.01
STX	0.13–1.07	0.9990	0.9980	≥0.03	≥0.09

**Table 2:** Minimum LOD and LOQ values expressed as µM STX eqv. (top row) and µg STX.2HCl eqv./kg (bottom row). TEFs reported by EFSA and FAO/WHO were both considered. Minimum LOQ values from AOAC official method 2005.06 are also shown.

	EFSA			AOAC 2005.06 *	FAO/WHO		
PST	TEF	LOD (µM STX eqv.) (µg STX.2HCl eqv./kg)	LOQ (µM STX eqv.) (µg STX.2HCl eqv./kg)	LOQ (µM STX eqv.) (µg STX.2HCl eqv./kg) ^†^	TEF	LOD (µM STX eqv.) (µg STX.2HCl eqv./kg)	LOQ (µM STX eqv.) (µg STX.2HCl eqv./kg)
dcGTX2,3	0.4	1.6 × 10^−3^ 4.8 × 10^0^	5.2 × 10^−3^ 1.5 × 10^1^	--- 2.8 × 10^1 ‡^	0.4	1.6 × 10^−3^ 4.8 × 10^0^	5.2 × 10^−3^ 1.5 × 10^1^
GTX1,4	1.0	1.6 × 10^−2^ 9.5 × 10^1^	5.4 × 10^−2^ 3.2 × 10^2^	--- 5.0 × 10^1^	1.0	1.6 × 10^−2^ 9.5 × 10^1^	5.4 × 10^−2^ 3.2 × 10^2^
C3,4	0.1	6.0 × 10^−4^ 5.4 × 10^0^	1.9 × 10^−3^ 1.7 × 10^1^	--- 7.3 × 10^2^	0.1	6.0 × 10^−4^ 5.4 × 10^0^	1.9 × 10^−3^ 1.7 × 10^1^
C1,2	0.1	3.0 × 10^−4^ 8.9 × 10^−1^	1.1 × 10^−3^ 3.3 × 10^0^	--- 9.3 × 10^1^	0.1	3.0 × 10^−4^ 8.9 × 10^−1^	1.1 × 10^−3^ 3.3 × 10^0^
dcSTX	1.0	5.0 × 10^−3^ 1.5 × 10^1^	1.8 × 10^−2^ 5.4 × 10^1^	--- 8.0 × 10^0^	0.5	2.5 × 10^−3^ 7.4 × 10^0^	9.0 × 10^−3^ 2.7 × 10^1^
dcNEO	0.4	9.2 × 10^−3^ 6.8 × 10^1^	3.0 × 10^−2^ 2.2 × 10^2^	--- ---	0.2	4.6 × 10^−3^ 3.4 × 10^1^	1.5 × 10^−2^ 1.1 × 10^2^
NEO	1.0	1.7 × 10^−2^ 1.3 × 10^2^	5.5 × 10^−2^ 4.1 × 10^2^	--- 4.0 × 10^1^	2.0	3.4 × 10^−2^ 2.5 × 10^2^	1.1 × 10^−1^ 8.2 × 10^2^
GTX6	0.1	4.0 × 10^−4^ 2.4 × 10^0^	1.5 × 10^−3^ 8.9 × 10^0^	--- ---	0.05	2.0 × 10^−4^ 1.2 × 10^0^	7.5 × 10^−4^ 4.5 × 10^0^
GTX2,3	0.6	2.4 × 10^−3^ 7.1 × 10^0^	7.2 × 10^−3^ 2.1 × 10^1^	--- 1.3 × 10^2^	0.6	2.4 × 10^−3^ 7.1 × 10^0^	7.2 × 10^−3^ 2.1 × 10^1^
GTX5	0.1	4.0 × 10^−4^ 1.2 × 10^0^	1.2 × 10^−3^ 3.6 × 10^0^	--- 2.7 × 10^1^	0.1	4.0 × 10^−4^ 1.2 × 10^0^	1.2 × 10^−3^ 3.6 × 10^0^
STX	1.0	2.8 × 10^−2^ 8.3 × 10^1^	9.4 × 10^−2^ 2.8 × 10^2^	--- 2.2 × 10^1^	1.0	2.8 × 10^−2^ 8.3 × 10^1^	9.4 × 10^−2^ 2.8 × 10^2^

Note: for epimer pairs, the highest TEF value was considered. * AOAC official method 2005.06 uses TEFs proposed by EFSA [14]. † AOAC Official Method 2005.06 refers to “µg/kg”. It was assumed this corresponds to µg STX.2HCl eqv./kg, based on the recommendation of expression of the results [14]. ‡ Minimum value presented in the extension of the validation of AOAC Official Method 2005.06 for dc-GTX2,3 [15,16].

**Table 3:** Concentration range for each toxin, in µg STX.2HCl eqv./g (number of independent replicates for each toxin, *n* ≥ 3); relative standard deviation (RSD), in percentage, for instrumental (instr.) and intermediate (interm.) precision. HorRat means Horwitz values, estimated for intermediate precision.

PST	Conc. (µg STX.2HCl eqv./g)	RSD _intsr._ (%)	RSD _interm._ (%)	HorRat
dcGTX2,3	0.03–1.08	≤0.8	≤9.8	0.5
GTX1,4	0.58–4.63	≤2.6	≤12.8	0.8
C3,4	0.02–0.58	≤2.6	≤14.7	0.9
C1,2	0.003–0.20	≤0.8	≤4.0	0.1
dcSTX	0.23–3.72	≤1.2	≤13.4	0.8
dcNEO	0.22–3.50	≤2.3	≤22.2	1.7
NEO	0.47–7.55	≤3.0	≤17.5	1.2
GTX6	0.03–0.48	≤3.6	≤6.6	0.4
GTX2,3	0.05–1.28	≤2.6	≤12.7	0.6
GTX5	0.008–0.26	≤0.8	≤7.2	0.3
STX	0.40–3.18	≤2.3	≤4.7	0.3

**Table 4:** Retention times (R_t_), in minutes, for each toxin and its by-products, using our chromatographic method. Identification of the necessary procedures for the detection and quantification of each toxin. Px: peroxide oxidation; Pe: Periodate oxidation.

R_t_ (Minutes)	Products	Separation—Quantification
4.6–4.7	By-products of GTX1,4 and C3,4	---
5.4–5.5	dcGTX2,3 (1st peak)	SPE-C18—Px
5.8–5.9	GTX1,4 and C3,4 (and 2nd peak of dcGTX2,3)	C3,4: SPE-C18—SPE-COOH, fraction 1—Pe GTX1,4: SPE-C18—SPE-COOH, fraction 2—Pe dcGTX2,3: SPE-C18—SPE-COOH, fraction 2—Pe *
6.2–6.4	By-products of NEO and GTX6	---
6.9–7.0	C1,2	SPE-C18—Px
7.3–7.4	dcSTX and dcNEO (1st peaks)	dcSTX: SPE-C18—Px dcNEO: SPE-C18—(SPE-COOH, fraction 3)—Pe
7.7–7.8	NEO and GTX6 (and 2nd peaks of dcSTX and dcNEO)	GTX6: SPE-C18—SPE-COOH, fraction 2—Pe NEO: SPE-C18—SPE-COOH, fraction 3—Pe dcNEO: SPE-C18—SPE-COOH, fraction 3—Pe dcSTX: SPE-C18—SPE-COOH, fraction 3—Pe *
8.3–8.4	GTX2,3 (and by-products of GTX1,4 and C3,4)	SPE-C18—Px
9.4–9.5	GTX5	SPE-C18—Px
9.9–10.0	STX (and by-products of GTX6 and NEO)	SPE-C18—Px

* This is not the recommended procedure for quantifying these toxins. Please see explanations in the text.

## Data Availability

Not applicable.

## Supplementary Materials

The following supporting information can be downloaded at: https://www.mdpi.com/article/10.3390/toxins14030179/s1, Details about determination of concentration in µM STX eqv. and µg STX.2HCl eqv./kg; Figure S1: Chromatograms of mix I (dcGTX2,3 + C1,2 + dcSTX), mix II (GTX2,3 + GTX5) and STX, after oxidation with peroxide; Figure S2: Chromatograms of mix III (GTX1,4 + NEO), mix IV (C3,4 + GTX6) and dcNEO, after oxidation with periodate; Figure S3: dcGTX2,3 standards after oxidation with periodate (black) or peroxide (pink).
