# Supplementary material for: Revisiting the HPLC-FLD Method to Quantify Paralytic Shellfish Toxins: C3,4 Quantification and the First Steps towards Validation
Source: Toxins (Basel). 2022 Feb 27;14(3):179. doi: 10.3390/toxins14030179 (PMC8949501; doi:10.3390/toxins14030179)
Supplement: Supplementary file 1 [file toxins-14-00179-s001.zip › toxins-1592709 supplementary.pdf]

# Supplementary Materials: Revisiting the HPLC-FLD Method to Quantify Paralytic Shellfish Toxins: C3,4 Quantification and the First Steps towards Validation

**Joana F. Leal and Maria L. S. Cristiano**

## Results

*Details about determination of concentration in µM STX eqv. and µg STX.2HCl eqv./Kg*

The following values were considered to estimate the LOD and LOQ in the above referred units, as presented in Table B1 of the document EURLMB SOP for the analysis of Paralytic shellfish toxins (PST) by precolumn HPLC-FLD according to OMA AOAC 2005.06.

$V_E$ = 10 mL

$m_H$ = 5 g

Dilution factor ($D_f$) for SPE-RP-C18 = 4.0 (for C1,2; GTX2,3; dcGTX2,3; GTX5; dcSTX; STX)

$D_f$ for SPE-RP-C18 plus SPE-COOH fraction 1 = 12.0 (for C3,4)

Df for SPE-RP-C18 plus SPE-COOH fraction 2 = 8.0 (for GTX1,4 and GTX6)

Df for SPE-RP-C18 plus SPE-COOH fraction 3 = 10.0 (for NEO and dcNEO)

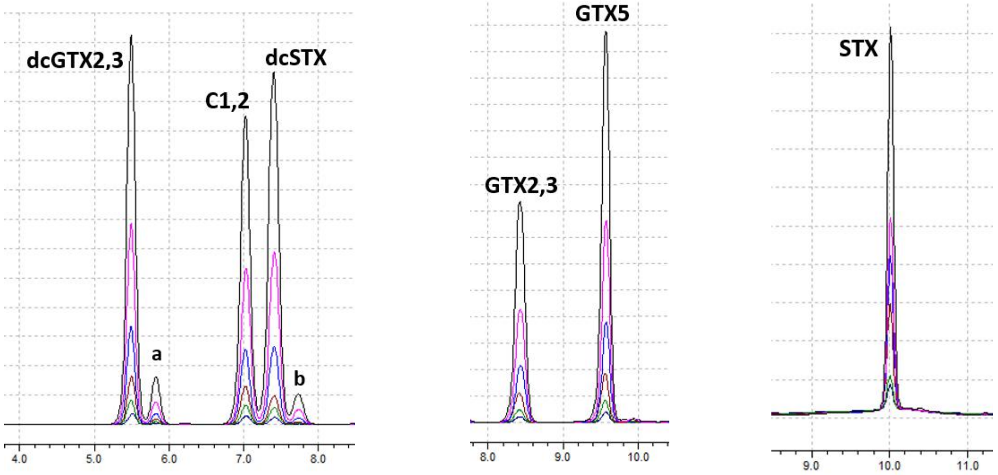


**Figure S1.** Chromatograms of mix I (dcGTX2,3 + C1,2 + dcSTX), mix II (GTX2,3 + GTX5) and STX, after oxidation with peroxide. **a** and **b** are oxidation products related to dcGTX2,3 and dcSTX, respectively.

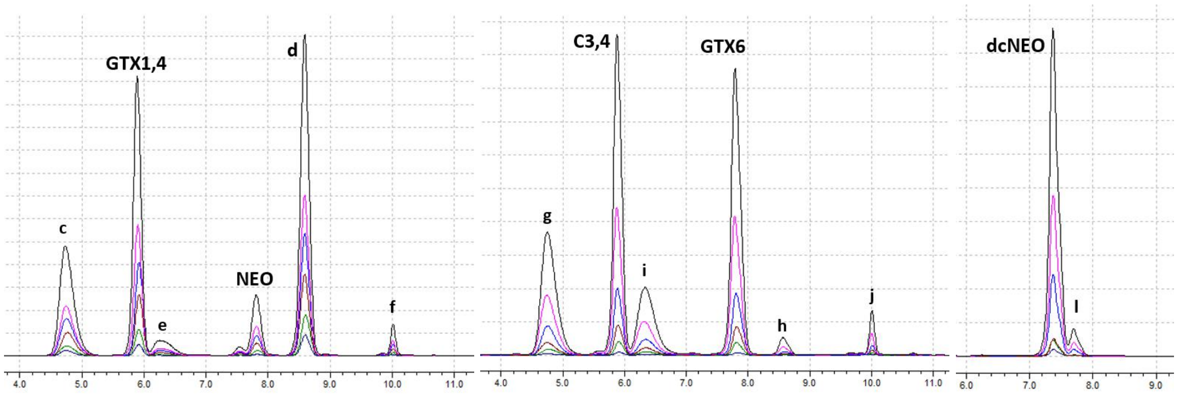


**Figure S2.** - Chromatograms of mix III (GTX1,4 + NEO), mix IV (C3,4 + GTX6) and dcNEO, after oxidation with periodate. **c** and **d** are oxidation products related to GTX1,4, while **e** and **f** correspond to oxidation products of NEO. **g** and **h** are oxidation products related to C3,4, while **i** and **j** correspond to oxidation products of GTX6. **g**, **h**, **i** and **j** products seem to correspond to the same products identified as **c**, **d**, **e** and **f**, respectively. **l** is an oxidation product of dcNEO.

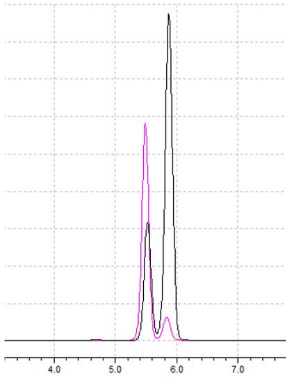

**Figure S3.** – dcGTX2,3 standard after oxidation with periodate (black) or peroxide (pink).
